# Yangyinqingfei decoction attenuates PM_2.5_-induced lung injury by enhancing arachidonic acid metabolism

**DOI:** 10.3389/fphar.2022.1056078

**Published:** 2022-11-16

**Authors:** Chunlan Tang, Yuqing Tang, Qinwen Wang, Donghui Chu, Jinyue Zhou, Yuping Zhou

**Affiliations:** ^1^ The Affiliated Hospital of Medical School, Ningbo University, Ningbo, Zhengjiang, China; ^2^ School of Medicine, Ningbo University, Ningbo, Zhengjiang, China; ^3^ Zhejiang Engineering Research Center of Advanced Mass Spectrometry and Clinical Application, Ningbo University, Ningbo, Zhengjiang, China

**Keywords:** Yangyinqingfei decoction, lung injury, proteomics, metabolomics, arachidonic acid metabolism

## Abstract

Yangyinqingfei Decoction (YYQFD), a traditional Chinese prescription, is well known in the treatment of diphtheria and lung-related diseases in clinic. However, whether it can be used to block the lung injury caused by air pollutant remains unclear. In the present study, the effect of YYQFD was addressed using a PM_2.5_-induced lung injury mice model. It was shown that YYQFD significantly improved pulmonary functions of mice exposed to PM_2.5_, the levels of IL-6, TNF-α and MDA were decreased while SOD levels were increased in serum and bronchoalveolar fluid. The potential mechanism of YYQFD was then delved using metabolomic and proteomic techniques. The protein-metabolite joint analysis showed that YYQFD regulated the biosynthesis of unsaturated fatty acids, linoleic acid and arachidonic acid metabolism, causing a significant decrement of pro-inflammatory mediator arachidonic acid with its downstream metabolites like 20-HETE, prostaglandin E_2_, accompanied by the up-regulation of PTGES2, GPX2 and CBR3 in lung tissue. These data were used to construct a regulatory metabolic network map in terms of the therapeutic role of YYQFD in PM_2.5_-induced lung injury, thereby provided a novel insight into potential application in the respiratory diseases caused by air pollutants.

## Introduction

Due to the rapid development of the economy and the increase of industrialization, more and more environmental pollution is occurring. Among them, air pollution is a vital part. According to the “Global Air Status 2019” report (Health Effects Institute, 2019), the number of deaths caused by long-term exposure to air pollutant in the world is as high as five million, among which 24% occurred in China. Presently, air pollution ranks fourth among all health risk factors, and premature deaths and health problems caused by air pollution cause losses of US $100–300 billion in China every year (Chen et al., 2013). The detrimental effect of PM_2.5_ is mainly determined by its size and composition. Due to its small particle size and large surface area, PM_2.5_ is easy to adsorb toxic and harmful substances, such as polycyclic aromatic hydrocarbons, heavy metal ions, SOx (sulfur oxides) and NOx (nitrogen oxides), *etc.* (Xing et al., 2016). Being the site for gas exchange and a barrier from the external environment, the respiratory system is exposed directly to high levels of PM_2.5_. Previous studies have shown that long-term exposure to PM_2.5_ can cause respiratory diseases including bronchitis and asthma, as well as cardiovascular diseases (Simoni et al., 2015; Maji et al., 2017; Li et al., 2018; Hayes et al., 2020). Therefore, an urgent task is to find effective drugs to prevent and treat respiratory diseases caused by PM_2.5_ and explore their mechanisms.

Yangyinqingfei Decoction (YYQFD) was first recorded in <*ChonglouYuyao*>. It consisted of Rehmannia Radix, Ophiopogonis Radix, Scrophulariae Radix, Glycyrrhizae Radix et Rhizoma, Menthae Haplocalycis Herba, Fritillariae Thunbergii bulbs, Moutan Cortex, and Paeoniae Radix Alba. With the function of nourishing and clearing the lungs, YYQFD is well known for the treatment of diphtheria. Nowadays, it is commonly used to acute pharyngitis, tonsillitis, and nasopharyngeal cancer treatment in clinic. Studies have reported that YYQFD is also used in combination with western medicine to treat stable chronic obstructive pulmonary disease (He, 2016; Fan et al., 2019). In the late 1980s, based on the original prescription, our affiliated hospital developed an in-hospital preparation of YYQFD with five Chinese medicinal materials including Ophiopogonis Radix, Rehmanniae Radix, Fritillariae Thunbergii bulbs, Glycyrrhizae Radix et Rhizome and Scrophulariae Radix, which is clinically used in the treatment of acute and chronic pharyngitis, pharyngeal neurosis, and chronic bronchitis. After years of clinical verification, this preparation showed good efficacy and fewer side effects.

A previous study has shown that YYQFD may have a protective effect on radiation-induced lung injury in rats by down-regulating the expression of MMP12 and TIMP1 (Li et al., 2014). However, there is still a lack of research on the mechanism of YYQFD in preventing and treating lung injury, and research is limited to a single herb on the treatment of lung injury or inflammation in the body. Scrophulariae Radix showed the protective effects on OVA-induced allergic airway inflammation *via* the suppression of NF-κB phosphorylation and the enhancement of the Nrf2/HO-1 signaling pathway (Jung et al., 2020). The active compound of Ophiopogonis Radix could inhibit migration and invasion in non-small cell lung cancer cells through enhancing the interaction between Axin and β-catenin (Zhang et al., 2021). In addition, Rehmannia Radix extract promoted apoptosis and inhibited proliferation of human lung fibroblasts cell line HFL1, and then arrested the progression of pulmonary fibrosis *via* inhibition on TGF-ß1 and Smad3 (Hu and Zhu, 2020). The efficacy of a single herb on associated lung disease provided a certain scientific basis for the further study of decoctions. Therefore, the intervention effect and mechanism of YYQFD in preventing and treating lung diseases caused by PM_2.5_ was investigated in this study.

Due to the effects of YYQFD in the clinical treatment of lung diseases, as well as the increasingly serious environmental pollution, the lung injury model induced by PM_2.5_ was duplicated in mice to explore the effect and mechanism of YYQFD in treating lung injury. The effects of YYQFD were observed through pulmonary histopathology, the levels of inflammatory factors IL-6 and TNF-α in bronchoalveolar fluid (BALF) and serum, as well as the level of SOD and MDA in BALF and serum. Also, the mechanism of YYQFD was further elaborated by metabolomics and proteomics. The present study could provide a better understanding of the therapeutic effect of YYQFD in the respiratory diseases caused by air pollutants.

## Material and methods

### Drugs and reagents

The Chinese herbs Ophiopogonis Radix, Rehmanniae Radix, FritillariaeThunbergii bulbs, Glycyrrhizae Radix et Rhizome and Scrophulariae Radix were provided by the Affiliated Hospital of Medical School of Ningbo University. PM_2.5_ samples were provided by the Ningbo Municipal Environmental Monitoring Center. The anti-GPX2 antibody, anti-CBR3 antibody, and anti-PTGES2 were purchased from ABclonal Technology (Wuhan, China). The anti-β-actin antibody was purchased from Santa Cruz Biotechnology (Santa Cruz, CA, United States). The HRP labeled sheep anti-rabbit IgG, sheep anti-mouse IgG reagents were purchased from Wuhan Boster Biological Company (Wuhan, China). The IL-6 and TNF-α assay kits were purchased from MultiSciences Biotechnology (Hangzhou, China). The SOD and MDA assay kits were purchased from Jiangsu enzyme-free Biotechnology (Yancheng, China).

### PM2.5 and YYQFD preparation

The PM_2.5_ quartz filter membrane was cut into small fragments of 1 cm*1 cm, and ultra-pure water was added. The mixture was oscillated twice with 30 min each. The eluents were combined and filtered with eight layers of sterile gauze, and the suspension containing PM_2.5_ was lyophilized for 24 h. PM_2.5_ fine particles were weighed and stored in the dryer.

The crude herbs of Ophiopogonis Radix, Rehmanniae Radix, Fritillariae Thunbergii bulbs, Glycyrrhizae Radix et Rhizome and Scrophulariae Radix were weighed according to the proportions for the YYQFD formula. 8-Fold (v/w) ultrapure water was added for a 30 min soak, the herb mixture was boiled for 2 h to collect the decoction. Then, fresh water was added to the herb mixture for another 1 h boiling decoction collection. The two decoctions were combined and filtered with eight layers of gauze. Enough ultra-pure water was added to the filtrate to make a total of 500 mL. This filtrate was then kept at −20°C.

### Animals

Male C57BL/6 mice (20 ± 2 g) were housed in a temperature-controlled room (22°C) with a 12-h light/dark cycle and were provided free access to laboratory chow and tap water. Male mice were selected for modeling because the tolerance of male mice was stronger than that of female mice, and there was no periodic physiological index fluctuation in male mice, with stable hormone levels and relatively stable enzyme activities. All of the animal protocols followed the “National Institutes of Health guide for the care and use of laboratory animals” and were approved by the Experimental Animal Ethics Committee of Ningbo University.

### Model establishment and drug treatment

45 mice were randomly divided into three groups with 15 mice each: control group, model group, and YYQFD group. Lung injury models were established by tracheal dripping of PM_2.5_ suspension (3 mg/kg) on the 16th, 24th, 32nd, and 39th day of modeling. The YYQFD group was given YYQFD *via* intragastric administration from the first day of modeling, and the other two groups were given the same amount of normal saline as control. The dose of YYQFD was 20 g/kg, which is converted from clinical dose of YYQFD (2.19 g/kg). At the end of modeling, the mice were sacrificed, and lung tissue, alveolar lavage, and serum were collected.

### Histologic assessment

The lung was harvested and fixed in buffered formaldehyde after lavage. The fixed lung was embedded in paraffin and performed the hematoxylin and eosin (H&E) staining for light microscopy. The histopathological score of lung injury in mice was performed using the method mentioned in the Report of the Official Symposium of the American Thoracic Society: Characteristics and Measurement of Experimental Acute Lung Injury in Animals (Matute-Bello et al., 2011). Twenty high-power fields (40 × 10) were randomly selected from the lung pathology sections stained with H&E, and each field was scored independently by blinded method. In addition, at least 50% of the alveoli were present in each field, visual fields dominated by the atmospheric tract or vascular lumen should be excluded. Detailed criteria for lung injury score were shown in Supplementary Table S1. The score = [(20×A) + (14×B) + (7×C) + (7×D) + (2×E)]/(view numbers ×100). The lung coefficient = wet lung weight (g)/body weight (kg) ×100%.

### Measurement of IL-6, TNF-α, SOD and MDA

The levels of IL-6 and TNF-α in serum and BALF, SOD and MDA in serum were measured using commercially available kits according to the manufacturer’s instructions. All samples were assayed in triplicate.

### Sample preparation for metabolomic analysis

Lung tissue samples were slowly thawed at 4°C, and pre-cooled methanol/acetonitrile/water solution (2:2:1, v/v) was added. The sample was eddy mixed and low-temperature sonicated for 30 min, followed by maintaining at −20°C for 10 min and centrifugation at 14,000 g and 4°C for 20 min. The supernatant was vacuum-dried, and 100 μL acetonitrile-water solutions (1:1, v/v) were added to dissolve the sample. Then it was centrifuged at 14,000 g and 4°C for 15 min, and the supernatant was sampled for analysis.

### Metabolomic detection by UHPLC-Q-TOF/MS

The samples were separated on an Agilent 1290 Infinity LC ultra-performance liquid chromatography (UHPLC) system with a HILIC column (Waters, ACQUITY UPLC BEH Amide 1.7 μm, 2.1 mm × 100 mm column). The column temperature, flow rate, and injection volume were set at 25°C, 0.5 mL/min, and 2 μL, respectively. The mobile phase included water with 25 mM ammonium acetate and 25 mM ammonia water (A) and acetonitrile (B). The gradient elution procedure was as follows: 0–0.5 min, 95%B; 0.5–7 min, B changes linearly from 95% to 65%, 7–8 min, B changes linearly from 65% to 40%, 8–9 min, B maintained at 40%, 9–9.1 min, B changes linearly from 40% to 95%; 9.1–12 min, B maintained at 95%.

The AB Triple TOF 6600 mass spectrometer was used to collect the MS and MS^2^ spectra of the samples. The ESI Source conditions after HILIC chromatographic separation are as follows: Ion Source Gas1: 60, Ion Source Gas2: 60, Curtain gas: 30, source temperature: 600°C, IonsSapary Voltage Floating: ±5500 V; TOF MS scan m/z range: 60–1000 Da, product ion scan m/z range: 25–1,000 Da, TOF MS scan accumulation time: 0.20 s/spectra, product ion scan accumulation time: 0.05 s/spectra. The secondary mass spectrum was obtained by information dependency acquisition, and the high sensitivity mode was adopted. Declustering potential (DP) and collision energy were set as ±60 V and 35 ± 15 eV, respectively. IDA is set as follows: isotopes within 4 Da, candidate ions to monitor per cycle: 10.

Peak alignment, retention time correction, and peak area extraction were performed using XCMS software. The data extracted by XCMS were firstly identified by metabolite structure and preprocessed. Principal component analysis (PCA) and orthogonal partial least squares discriminant analysis (OPLS-DA) were carried out using SIMCA-P 15.0.

### Sample preparation for proteomics analysis

Protein was extracted from lung tissue using the SDT (4% (W/V) SDS, 100 mM Tris/HCl with pH7.6, 0.1 M DTT) lysis method, and then quantified by the BCA method. The protein samples were digested by trypsin using Filter Aided Proteome Preparation (Wiśniewski et al., 2009), and the peptide segment was desalted using a C18 column. After lyophilized, the peptide was dissolved in 40 μL 0.1% formic acid solution and quantified (OD280). Then, the quantified samples were labeled with TMT.

RP grading: Each labeled peptide was mixed in equal quantities and graded using the high pH reversed-phase peptide fractionation Kit. The column was equilibrated with acetonitrile and 0.1% trifluoroacetic acid, and the mixed labeled peptides were desalted. At last, the column-bound peptides were gradient eluted with a high pH acetonitrile solution with increasing concentrations. Each eluted peptide sample was lyophilized and dissolved with 12 μL 0.1% trifluoroacetic acid. The concentration of the peptide was determined by OD280. SCX grading: Each group of labeled peptides was mixed and graded using Akta Purifier 100. Buffer solution A (pH 3.0) consisted of 10 mM KH_2_PO_4_ and 25%ACN. Buffer solution B (pH 3.0) consisted of 10 mM KH_2_PO_4_, 500 mM KCl and 25%ACN. The chromatographic column was balanced with liquid A, and the sample was loaded from the sampler to the column for separation at a flow rate of 1 mL/min. The gradient of liquid phase is as follows: 0–25 min, the linear gradient of liquid B is from 0% to 10%; 25–32 min, the linear gradient of liquid B ranged from 10% to 20%; 32–42 min, the linear gradient of liquid B ranged from 20% to 45%; 42–47 min, the linear gradient of liquid B ranged from 45% to 100%; 47–60 min, liquid B was maintained at 100%; after 60 min, liquid B was reset to 0%. During the elution process, the absorbance value of 214 nm was monitored, and the eluted components were collected every 1 min. After lyophilized, the C18 Cartridge was used for desalination.

### Proteomics detection by TMT

The samples were separated by HPLC liquid phase system Easy NLC with nanolitre flow rate. Buffer solution A was 0.1% formic acid aqueous solution, and buffer solution B was 0.1% formic acid acetonitrile aqueous solution (the concentration of acetonitrile was 84%). The chromatographic column was balanced with 95% A, and the sample was loaded from the automatic sampler to the loading column (Thermo Scientific Acclaim Pepmap100, 100 μm*2 cm, Nanoviper C18). The samples were separated by a Thermo scientific EASY column (10 cm, ID75 μm, 3 μm, C18 A2) with a flow rate 300 nL/min.

After chromatographic separation, the samples were analyzed by Q Exactive mass spectrometer. The positive ion is adopted, the scanning range of the mother ion is 300–1,800 m/z, and the resolution of primary mass spectrometry is 70,000 at 200 m/z. The precursors were selected for HCD MS^2^ analysis with an automatic gaining control target of 1e^6^, maximum injection time of 50 ms, dynamic exclusion 60.0 s, isolation window of 2 m/z, and normalized collision energy of 30 eV. Mascot 2.2 and Proteome Discoverer 1.4 were used for database identification and quantitative analysis.

### Western blot analysis

Proteins were subjected to SDS-PAGE with a 12% running gel and were then transferred to a polyvinylidene fluoride membrane with different antibodies (1:1000) at 4°C for 12 h and then with a second antibody (1:2000) for 1.5 h. The immunofluorescence bands were detected using the Tanon 4200 SF multifunctional chemiluminescence instrument (Shanghai, China).

### Statistical analysis

Metabolomic data were analyzed using SIMCA-P software, and PCA and OPLS-DA were selected for statistical analysis. Other data were analyzed using SPSS 16.0 statistical software, and the measurement data were compared between groups using *t*-test or one-way analysis of variance. *p* < 0.05 was considered significant.

## Results

### YYQFD alleviated PM2.5-induced pathological injury of lung tissue

**FIGURE 1:**
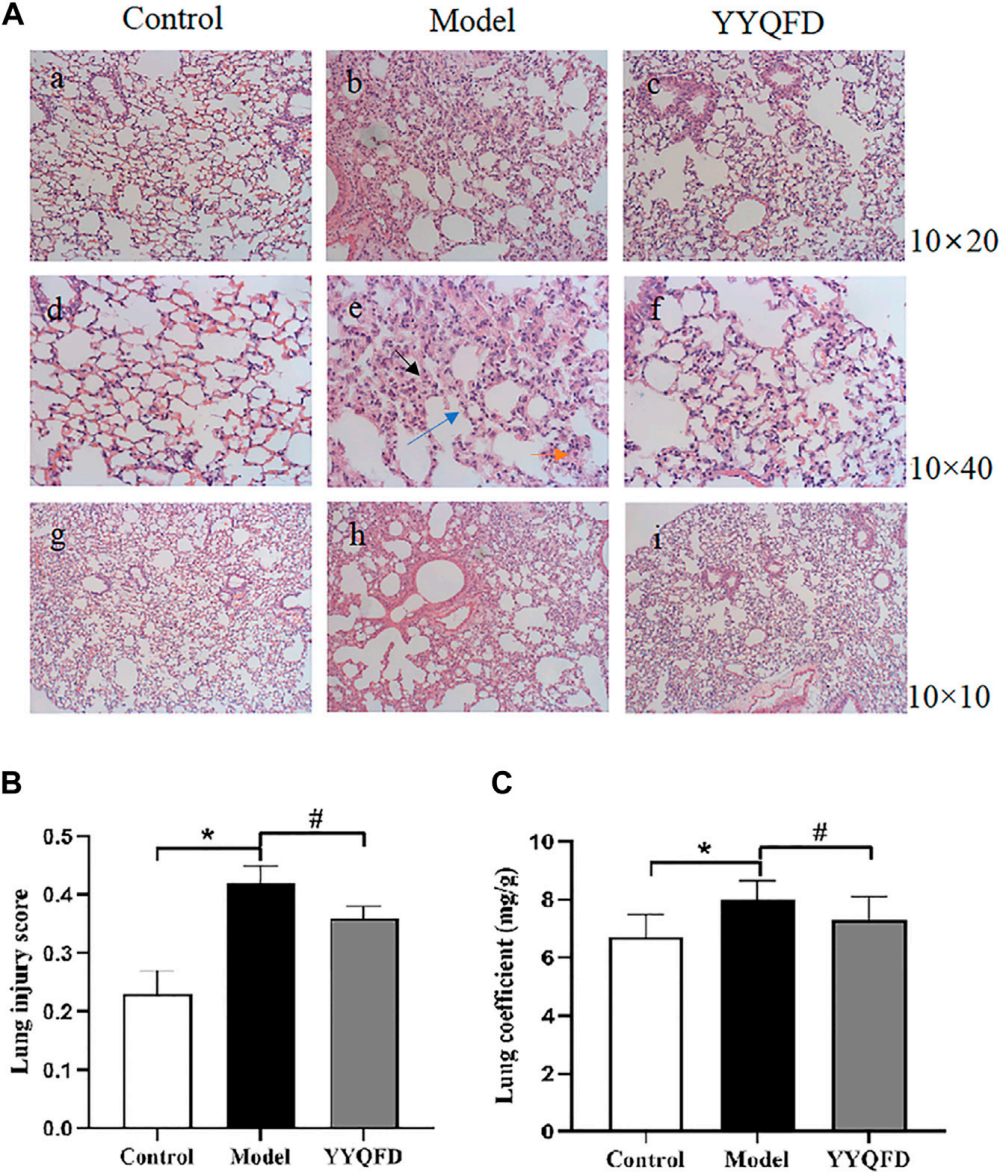
Histopathological changes of lung tissues **(A)** Pulmonary tissue slices stained by H&E of each group (Bars, a, b, c ×200; d, e, f ×400; g, h, i ×100), black arrows indicated thickening of alveolar walls, blue arrows indicated alveolar collapse, and orange arrows indicated inflammatory cell infiltration **(B)** Lung injury score of mice **(C)** Lung coefficients of mice. ^*^ *p* < 0.05 vs. control group, ^#^ *p* < 0.05 vs. model group.

Pathological staining and pathological scoring were performed to clarify the protective effect of YYQFD on PM_2.5_-induced lung injury. According to the results of H&E staining, it was found that the structure of the lung tissue in the model group was severely damaged. The thickness of alveolar walls significantly increased, and the alveoli collapsed and were disorganized with numerous neutrophils infiltration (Figure 1Ab, e, h). Then, a combined pathological score of lung tissue (Figure 1B) and lung coefficients (Figure 1C) was generated, which revealed a markedly increment in the model group compared to the control group, indicating that PM_2.5_ induced acute pulmonary injury and inflammation in mice. Whereas, the above symptoms of the structure of lung tissue were significantly improved in the YYQFD groups (Figure 1Ac, f, i), and pathological score and lung coefficients is lower in the YYQFD groups compared with the model group (Figures 1B, C). Thus, the results demonstrated that YYQFD could effectively alleviate PM_2.5_-induced acute lung injury.

### YYQFD ameliorated PM2.5-induced inflammatory responses and oxidative stress

**FIGURE 2:**
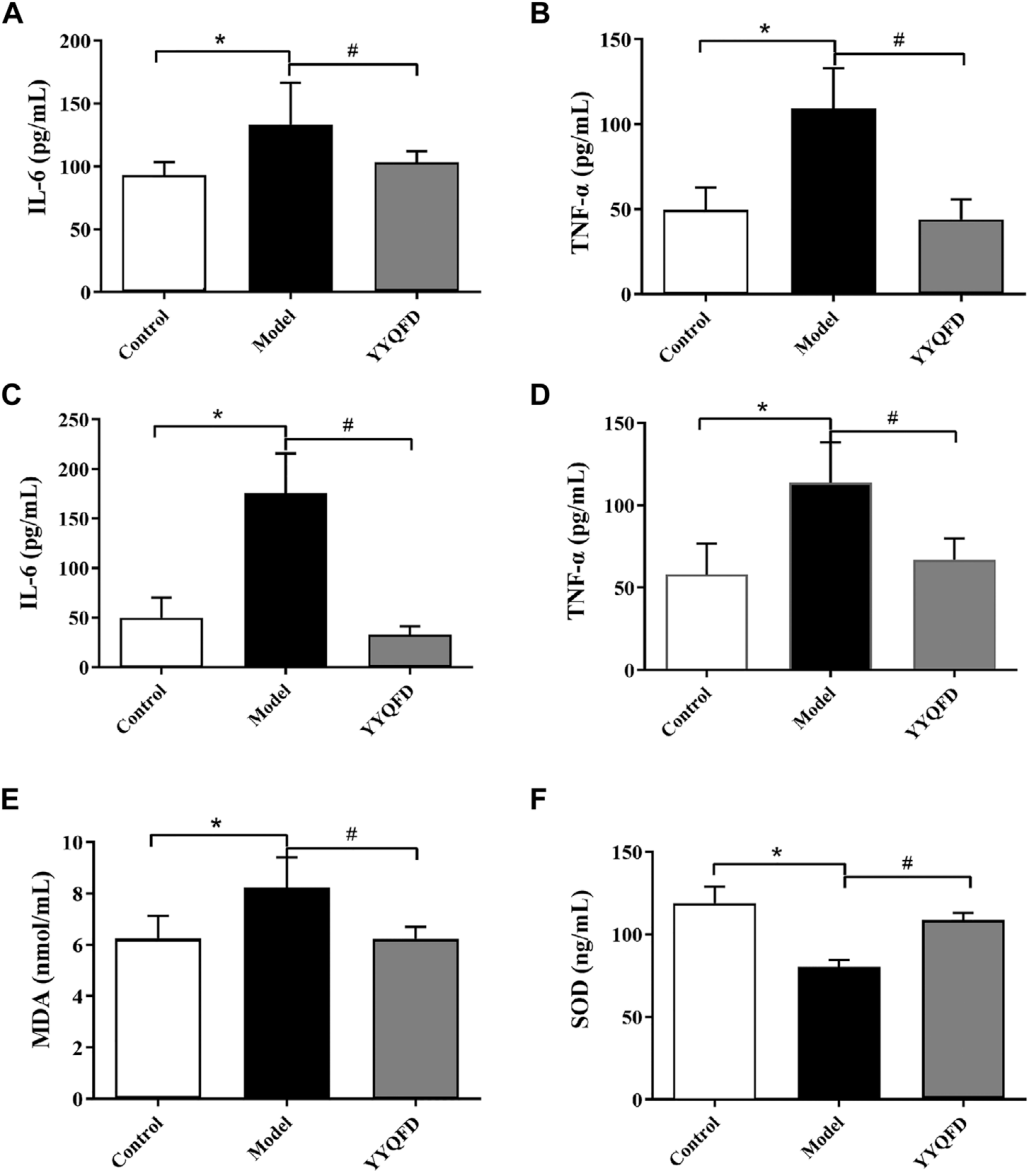
The IL-6, TNF-α, MDA and SOD levels in BALF and serum measured with ELISA kits **(A)** and **(B)** IL-6 and TNF-α levels in BALF **(C)** and **(D)** IL-6 and TNF-α levels in serum **(E)** and **(F)** MDA and SOD levels in serum. ^*^ *p* < 0.05 vs. control group, ^#^ *p* < 0.05 vs. model group.

The inflammatory cytokines could affect the lung inflammatory responses, thus the cytokines levels in BALF and serum were measured. The levels of IL-6 and TNF-α in BALF and serum both significantly increased in the model group compared with the control group, whereas YYQFD could significantly decrease IL-6 and TNF-α levels in BALF and serum (Figures 2A–D). MDA and SOD levels in serum were also measured with ELISA kits. The level of MDA in serum significantly increased in the model group compared with the control group, whereas YYQFD significantly decreased the MDA level (Figure 2E). On the contrary, the level of SOD in serum significantly decreased in the model group and YYQFD increased the SOD level (Figure 2F). These results suggested that the inflammatory response and oxidative stress level was simulated by PM_2.5_ and YYQFD can effectively inhibit the inflammatory response and reduce the level of oxidative stress.

### Effect of PM2.5 exposure and YYQFD on metabolomic profiles in lung tissue

**FIGURE 3:**
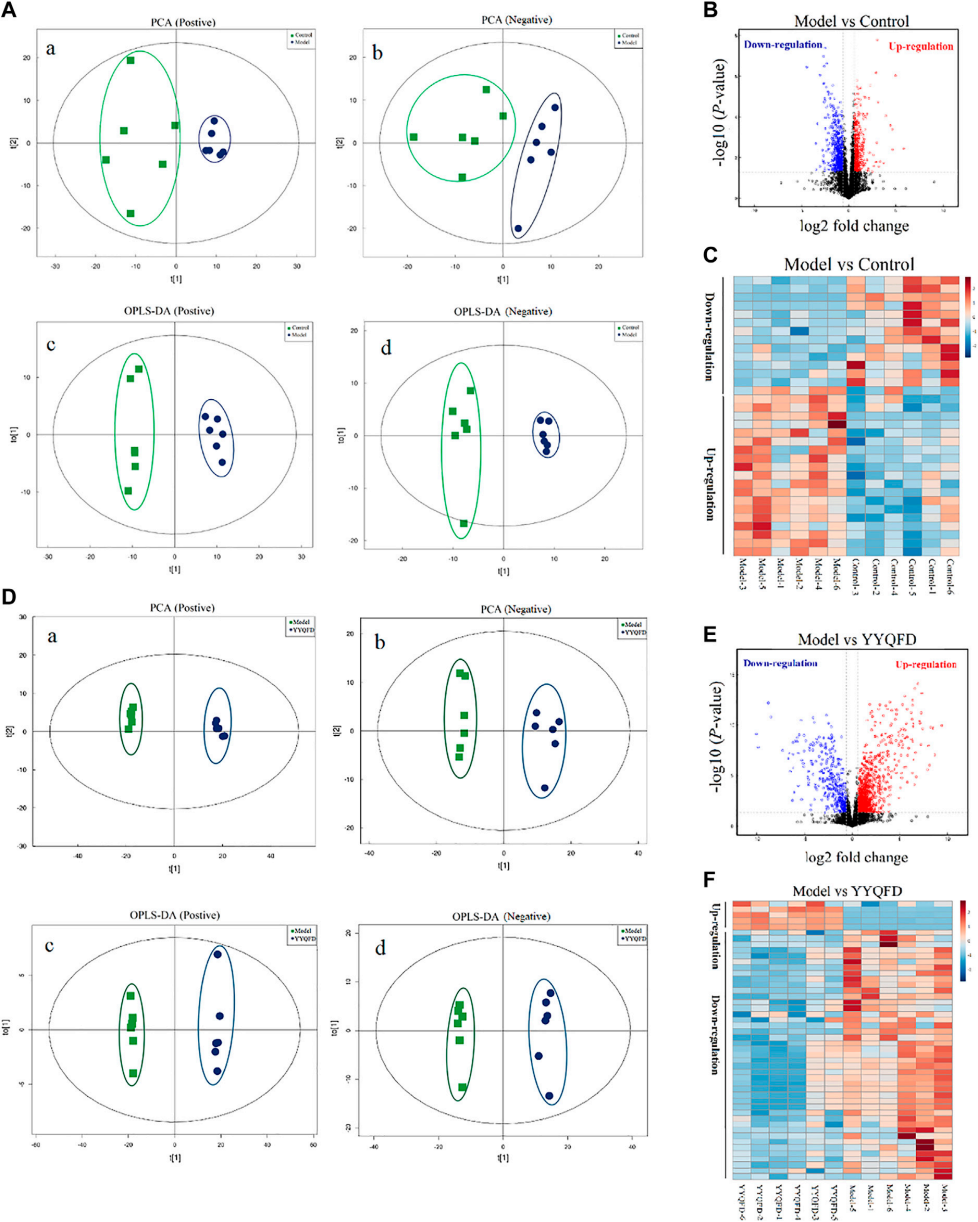
The effect of PM_2.5_ exposure and YYQFD intervention on metabolomic profiles in lung tissues **(A,B and C)** Model group vs. Control group **(D,E and F)** YYQFT group vs. Model group (Aa)and (Da) PCA scatter map of lung metabolites in positive ion mode (R^2^X = 0.517 and 0.751) (Ab)and (Db) PCA scatter map of lung metabolites in negative ion mode (R^2^X = 0.539 and 0.522) (Ac)and (Dc) OPLS-DA scatter map of lung metabolites in positive ion mode (Q^2^ = 0.876 and0.990) (Ad) and (Dd) OPLS-DA scatter map of lung metabolites in negative ion mode (Q^2^ = 0.817 and 0.963) **(B)** and**(E)** Volcano plots of detected metabolites **(C)** and **(F)** Heatmap analysis of significantly differentially expressed metabolites.

Metabolic profiling of mice lung tissue was analyzed using UHPLC Q-TOF/MS to examine the effect of PM_2.5_ exposure and YYQFD intervention on pulmonary metabolomic change. PCA and OPLS-DA plots showed a clear separation between the model group and the control group, indicating that the induction of PM_2.5_ displayed a metabolic change in lung tissues (Figure 3A). A total of 16,810 metabolites were detected between the model group and the control group (Figure 3B). Among them, 481 metabolites were identified, with 13 significantly downregulated and 20 upregulated (*p* < 0.05 and VIP > 1) (Supplementary Table S2). These metabolites were mainly lipids and lipid molecules, amino acids, and carbohydrates (Figure 3C). Similarly, PCA and OPLS-DA plots also showed a clear separation between the YYQFD group and the model group (Figure 3D). 19,509 metabolites were detected between the model group and the YYQFD group (Figure 3E). A total of 492 metabolites were identified, with 47 significantly downregulated and 4 upregulated (*p* < 0.05 and VIP > 1) (Supplementary Table S3) (Figure 3F). These metabolites mainly included fatty acids, amino acids, peptides, and analog.

**FIGURE 4:**
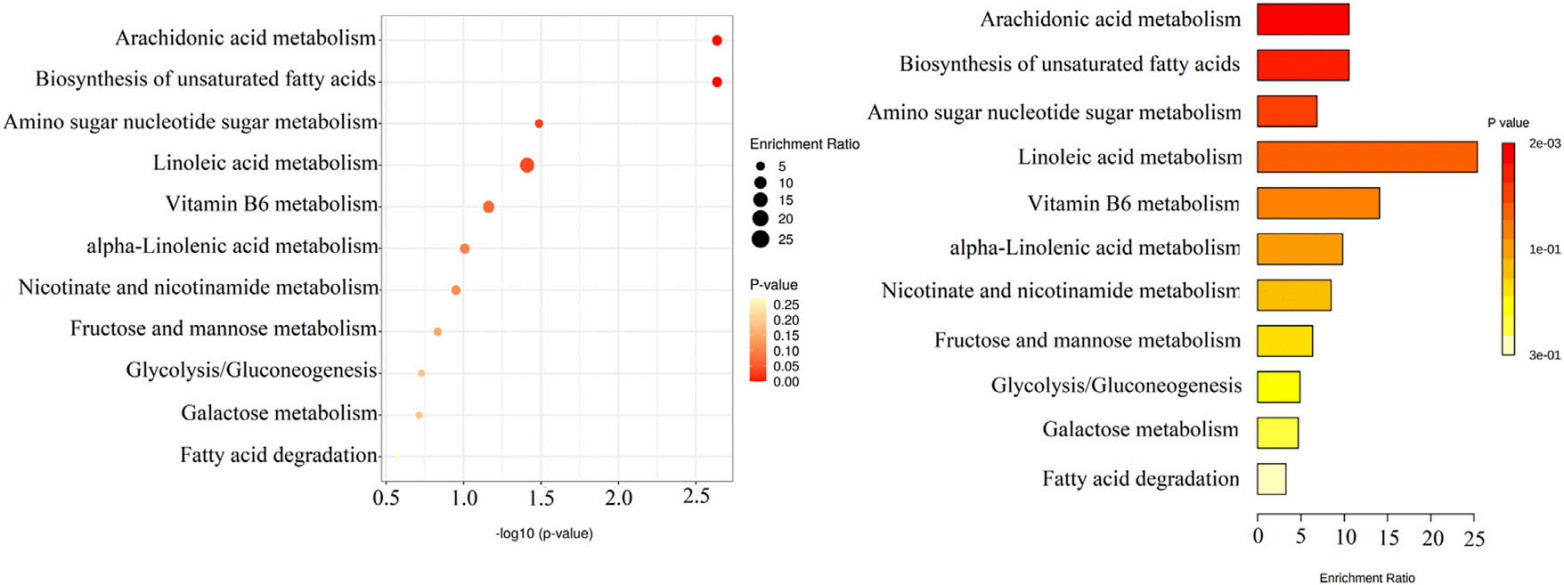
KEGG enrichment pathways of 13 differential metabolites.

Overall, 13 metabolites were significantly up-regulated under the PM_2.5_ exposure and further down-regulated under YYQFD treatment, including arachidonic acid (AA), 20-hydroxyeicosatetraenoic acid (20-HETE), prostaglandin E2 (PGE2), lecithin, linoleic acid (LA), α-linolenic acid (α-LA), eicosapentaenoic acid (EPA), γ-linolenic acid (γ-LA), N-acetylmannosamine, 4-pyridoxic acid, l-palmitoylcarnitine, 1-methylnicotinamide and α-d-glucose. Interestingly, YYQFD treatment resulted in significant changes in lipid metabolism, including arachidonic acid metabolism, biosynthesis of unsaturated fatty acids, and linoleic acid metabolism (Figure 4).

### Effect of PM2.5 exposure and YYQFD on proteomic profiles in lung tissue

**FIGURE 5:**
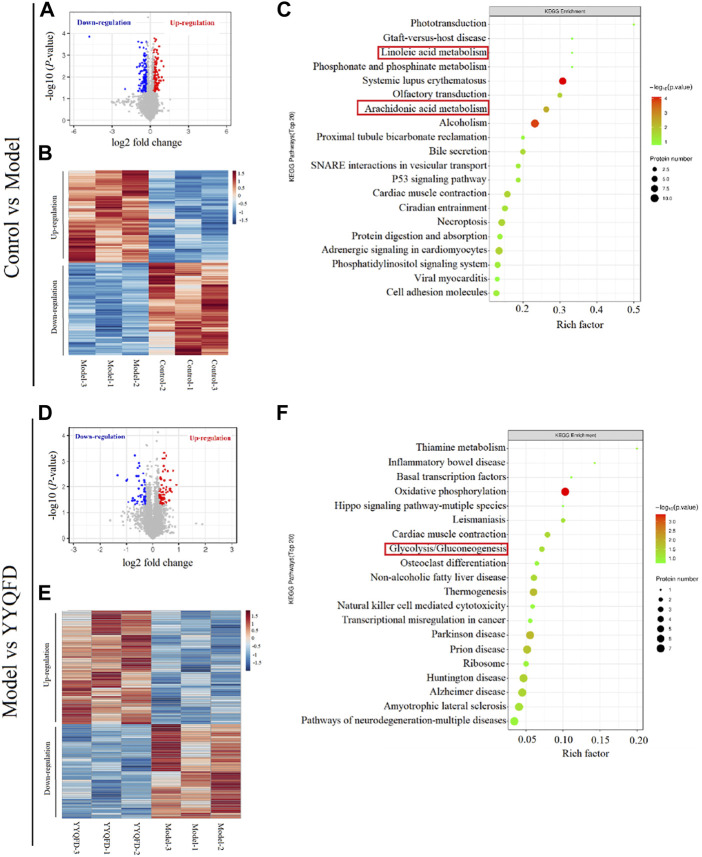
The effect of PM_2.5_ exposure and YYQFD intervention on proteomic profiles in lung tissue **(A,B and C)** Model group vs. Control group **(D,E and F)** YYQFT group vs. Model group **(A)** and** (D)** Volcano plots of detected protein **(B)** and** (E)** Heatmap analysis of significantly differentially expressed proteins **(C)** and** (F)** KEGG enrichment pathways of differential proteins.

The proteomic analysis was also carried out to quantify the change in proteome under the PM_2.5_ exposure and YYQFD intervention. The volcano plots of the proteins showed 5854 proteins detected in the model group, with 172 significantly downregulated and 171 upregulated after PM_2.5_ exposure (*p* < 0.05 and fold change>1.2 or <0.83) (Figure 5A). Among them, 48 proteins with fold change >1.5 were listed in Supplementary Table S4. The heatmap also showed the clustering of proteins between the PM_2.5_ and the control group (Figure 5B). In addition, 54 proteins significantly downregulated and 65 upregulated based on the YYQFD treatment (Figure 5D). Among them, 20 proteins with fold change >1.5 were listed in (Supplementary Table S5). The heatmap also showed the clustering of proteins between the PM_2.5_ and the YYQFD group (Figure 5E). Interestingly, these proteins enriched in arachidonic acid metabolism, linoleic acid metabolism and glycolysis/gluconeogenesis (Figures 5C, F). This was almost consistent with the pathways of differential metabolite enrichment.

### Protein-metabolite joint analysis

Protein-metabolite joint analysis was carried out to obtain the overall profiling of the effect under the PM_2.5_ exposure and YYQFD intervention. Arachidonic acid metabolism, linoleic acid metabolism, biosynthesis of unsaturated fatty acids and Glycolysis/Gluconeogenesis pathways were significantly regulated. Intriguingly, arachidonic acid metabolism, linoleic acid metabolism, and biosynthesis of unsaturated fatty acids attracted our interest.

**FIGURE 6:**
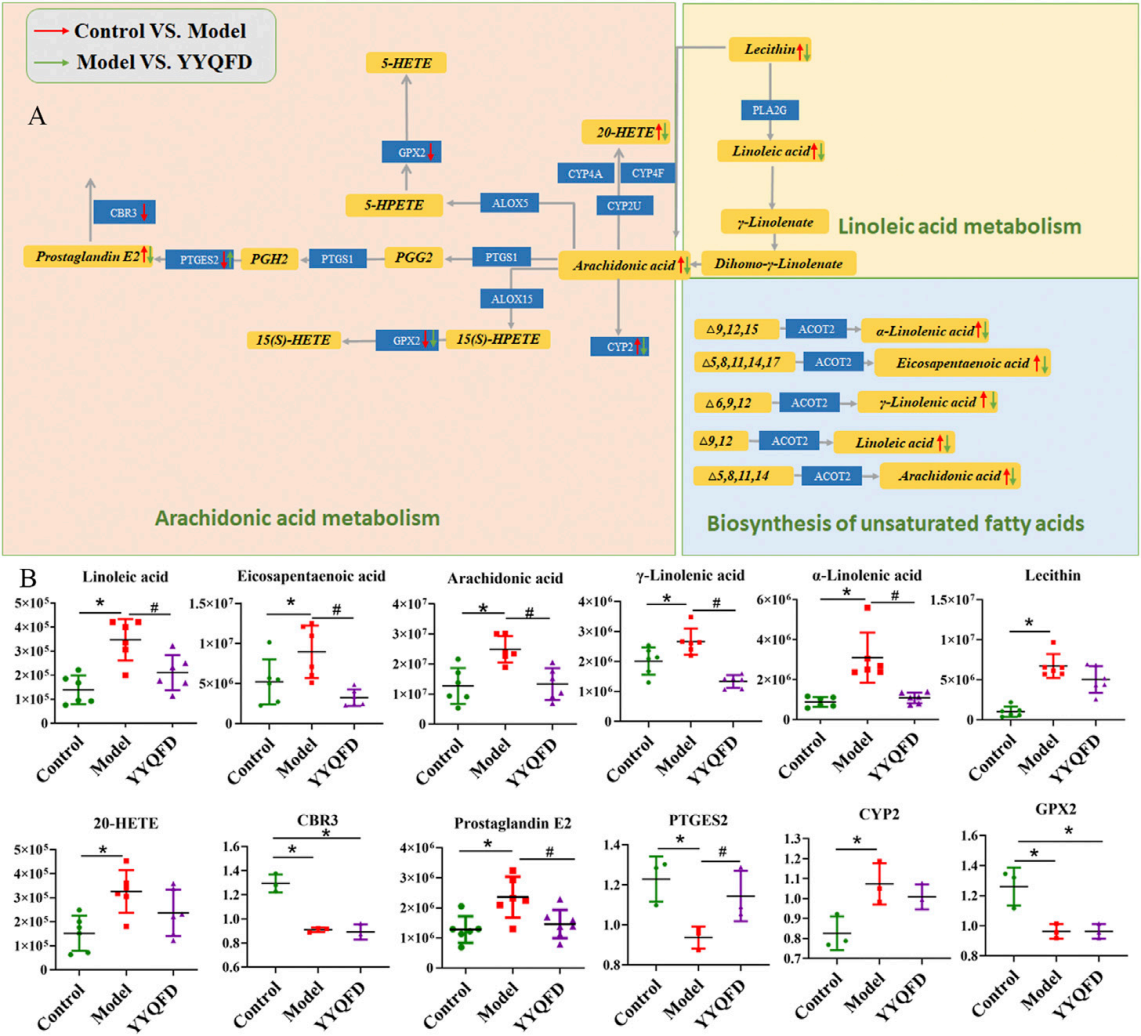
Arachidonic acid metabolism, linoleic acid metabolism and biosynthesis of unsaturated fatty acids pathways **(A)** and the content of significantly changed metabolites and proteins among the control, model and YYQFD group **(B)**.

**FIGURE 7:**
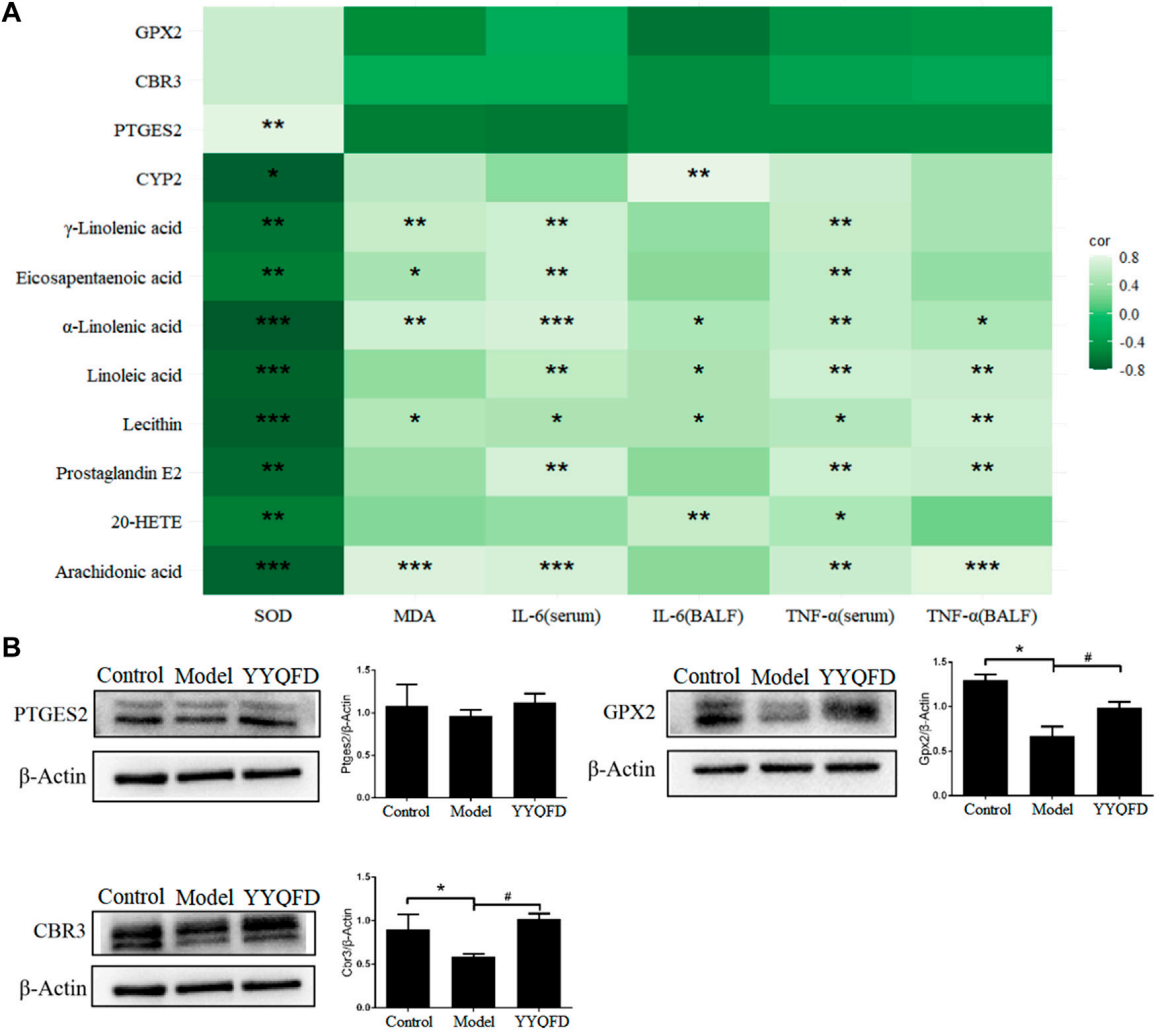
Correlation of differential metabolites and proteins with IL-6, TNF-α, MDA and SOD **(A)**, *p < 0.05, **p < 0.01, ***p < 0.001; the expression of PTGES2, GPX2 and CBR3 proteins in lung tissue of mice among the control, model and YYQFD group **(B)**, ^*^ *p* < 0.05 vs. control group, ^#^ *p* < 0.05 vs. model group.

In these three pathways, eight metabolites were significantly up-regulated in the model group, including AA, 20-HETE, PGE2, lecithin, LA, α-LA, EPA, and γ-LA. It is also shown that three proteins were significantly down-regulated in the model group, including PTGES2, CBR3, and GPX2, and CYP2 was down-regulated in the model group. These metabolites and proteins may be biomarkers for PM_2.5_-induced lung injury. Among them, the levels of AA, 20-HETE, PGE2, lecithin, LA, α-LA, EPA, γ-LA, PTGES2 and CYP2 levels recovered in YYQFD group (Figure 6). Although proteomic results showed no significant recovery of proteins CBR3 and GPX2 levels in the YYQFD group, their levels were up-regulated in the WB test (Figure 7B). These metabolites and proteins may be the targets of YYQFD to treat PM_2.5_-induced lung injury. Furthermore, the correlation analysis between these biomarkers and biochemical parameters was carried out (Figure 7A). Pearson correlation analysis was used, and there is a linear correlation between the two variables when *p* < 0.05. The value of the correlation coefficient is between −1 and 1, and the closer the value is to 1, the stronger the correlation is. Interestingly, the levels of these eight metabolites decreased with the increase of SOD levels, and increased with the decrease of MDA, IL-6, and TNF-α level. On the contrary, the levels of proteins GPX2, CBR3, and PTGES2 increased with the increase of SOD levels and decreased with the decrease of MDA, IL-6, and TNF-α level.

## Discussion

**FIGURE 8:**
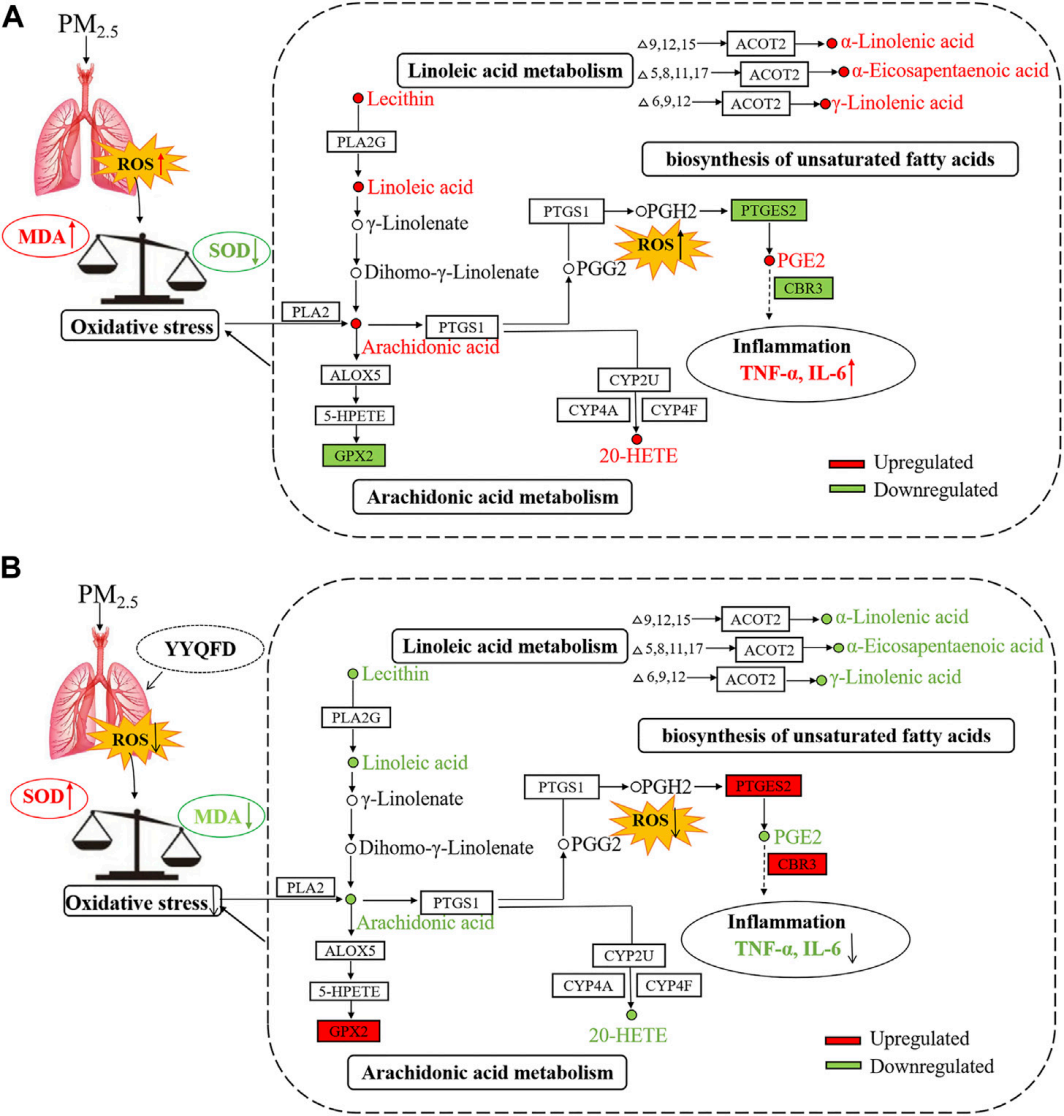
The mechanism diagram of the lung injury in mice caused by PM_2.5_ **(A)** and YYQFD repairing the lung injury in mice caused by PM_2.5_ **(B)**.

In this study, PM_2.5_ was given to mice through intra-tracheal instillation *in vivo*, and it was shown that PM_2.5_ exposure impaired the function of lung *via* inducing remarkable inflammation and oxidative stress. Then the effect of YYQFD on PM_2.5_-induced pulmonary injury was explored. The results showed that YYQFD could attenuate inflammation and oxidative stress by decreasing the histopathological score of lung injury, lowering the concentrations of IL-6 and TNF-α in serum and BALF, and decreasing the concentration of MDA and increasing the concentration of SOD in serum. Furthermore, metabolomics and proteomics were used to clarify the mechanism of YYQFD. Our results revealed the arachidonic acid metabolism, linoleic acid metabolism, and biosynthesis of unsaturated fatty acids pathways were significantly changed. Eight metabolites and four proteins were significantly up/down-regulated in the model group were recovered in the YYQFD treated group, including AA, 20-HETE, PGE2, lecithin, LA, α-LA, EPA, γ-LA, PTGES2, CBR3, CYP2, and GPX2. This indicated that these metabolites and proteins may be biomarkers for PM_2.5_-induced lung injury and also can be as the targets of YYQFD intervention. We also found that the levels of these eight metabolites decreased with the increase of SOD levels, and increased with the decrease of MDA, IL-6 and TNF-α level. On the contrary, the levels of proteins GPX2, CBR3, and PTGES2 were increased or decreased with the increase of SOD level and the decrease of MDA, IL-6, and TNF-α level, respectively. As a result, above mechanisms were summarized in a regulatory metabolic network map (Figure 8).

Previous studies have reported that acute exposure to low-dose PM_2.5_ induces inflammation, oxidative stress in the lung, and impairment of pulmonary function (Riva et al., 2011; Li et al., 2015). Therefore, PM_2.5_ was used to establish the pulmonary injury model in mice in this study. Consistent with previous studies, intratracheal instillation of PM_2.5_ induced acute pulmonary injury, indicated by a higher lung injury score. The increased infiltration of inflammatory cells into the interstation was observed after PM_2.5_ instillation, mainly with neutrophils. Also, alveolar wall thickening, alveolar collapse, and structural disorder were also observed. We also found that the levels of IL-6 and TNF-α increased in BALF and serum. PM_2.5_ has been reported to induce the production of reactive oxygen species (ROS) in neutrophils of asthmatic patients, which is involved in neutrophil activation and pulmonary injury (Sierra-Vargas et al., 2009). In this study, an increase of MDA levels and a decrease of SOD levels were observed. These results suggested that lipid peroxidation in mice increased after PM_2.5_ instillation, further causing an imbalance between oxidation and antioxidant systems. The administration with YYQFD significantly ameliorated these disorders with a reduction in the IL-6, TNF-α and MDA levels, an increase in the SOD levels in BALF, and a reduction of IL-6 and TNF-α in serum.

The harmful effects of PM_2.5_ on the human body are closely related to lipid metabolism. Long-term exposure to PM_2.5_ can induce dyslipidemia (Ramanathan et al., 2016), vascular inflammation (Sun et al., 2005) and fat dysfunction (Wan et al., 2014). Plasma lipid metabolic profile after PM_2.5_ exposure was described in past studies (Breitner et al., 2016). However, as the main barrier following exposure to a high level of PM_2.5_, pulmonary metabolomics has not been carefully studied. In this study, the metabolomics of lung tissue from mice instilled with PM_2.5_ *in vivo* was examined. At the same time, the proteomics of lung tissue was also examined to avoid the data loss of single omics and data problems caused by noise and other factors.

The metabolomics profile revealed that PM_2.5_ instillation mainly increased lipid metabolisms in lung tissue. The increased levels of AA, 20-HETE, lecithin, PGE2, α-LA, LA, γ-LA, and EPA were observed after PM_2.5_ instillation. These eight metabolites were mainly involved in the arachidonic acid metabolism, linoleic acid metabolism, and biosynthesis of unsaturated fatty acids pathways. As an essential polyunsaturated fatty acid, AA is widely distributed in the human body. But there is very little free AA in the body, mainly in the form of binding to the hydroxyl group on the glycerophospholipid. When oxidative stress occurred in the body, the body would promote the release of AA by enhancing the activity of phospholipase A2 (Tao and Fu, 2019). AA was mainly used to synthesize pro-inflammatory factors. After pro-inflammatory factors are bounded to inflammatory cell receptors, they could activate intracellular inflammatory signal transduction, and promote the synthesis and release of inflammatory factors such as TNF-α and interleukins, thereby amplifying inflammation (Lewis et al., 1990). This was consistent with the increase in TNF-α and IL-6 levels observed after PM_2.5_ instillation. The released AA could be metabolized by the cyclooxygenase (COX) pathway to generate prostaglandins and thromboxanes, by the lipoxygenase (LOX) pathway to generateperleukotrienes and hydroxylated eicosatetraenoic acid, and by the cytochrome P450 enzyme (CYP450) pathway to produced hydroxylated eicosatetraenoic acid and epoxidized eicosatetraenoic acid. These three pathways of AA were closely related to lung injury. Mice deficient in COX-2 could attenuate LPS-induced inflammatory response and the resultant acute lung injury by regulation of LPS-induced levels of cytokines/chemokines (Nelin et al., 2022). 12/15-LOX was shown to be involved in the regulation of eicosanoid production in the respiratory system using knockout mice model. Moreover, the deficiency of 12/15-LOX could also affect the expression of other enzymes (COX-2 and 5-LOX) involved in metabolism of AA, and elevated concentration of PGE2 in BALF of 12/15-LOX knockout mice were also observed (Sacharzewska et al., 2016). When AA was metabolized through the COX and LOX pathways, many reactive oxygens would be produced, thereby aggravating oxidative stress (Wang et al., 2019). As a metabolite of AA, PGE2 was an important pro-inflammatory factor in the arachidonic acid metabolism pathway. It could regulate the differentiation of immune cells and the expression of cytokines during inflammation, thereby increasing the occurrence of inflammation (Das, 2021). In addition, 20-HETE was an important product of CYP450 catalyzing the production of AA. Previous studies had reported that 20-HETE could regulate the activity of nitric oxide synthase (eNOS) in pulmonary artery endothelial cells and dilates pulmonary arteries by increasing the release of nitric oxide (Cheng et al., 2008; Cheng et al., 2012). And hepatic CYP1A2 have been reported that it protects against hyperoxic lung injury by decreasing lipid peroxidation and oxidative stress *in vivo*, F2-isoprostanes that are formed during hyperoxia exposure may undergo detoxification by CYP1A2 to non-toxic metabolites. And in CYP1A2 knockout mice, the compound would accumulate and lead to increased lung injury (Lingappan et al., 2014; Lingappan et al., 2017). These results indicated that the oxidative stress in the mice after PM_2.5_ instillation led to the release of AA. Excessive AA generated more PGE2. Microsomal prostaglandin E synthase-1 (mPGES-1), cytosolic PGES (cPGES), and microsomal prostaglandin E synthase-2 (mPGES-2) are three known PGE2 synthases. PGE2 is synthesized by several pathways. One is to generate PGE2 by catalyzing COX-1 and COX-2-derived prostaglandin H2 (PGH2) through mPGES-1. Secondly, cPGES encoded by Ptges3 gene can convert PGH2 to PGE2. Third, PGE2 can be generated by mPGES-2 encoded by Ptges2 gene. Proteomics results showed that the PTGES2 level was significantly reduced after PM_2.5_ instillation, and the decreased level of PTGES2 was also observed in WB experiment, indicating that excessive AA might convert to PGE2 by mPGES-2. The decrease in PTGES2 expression level might be caused by being consumed in the process of catalyzing the redox process of PGH2 to PGE2. Furthermore, PGE2 promoted the synthesis and secretion of inflammatory factors, leading to increased inflammation in mice. Besides, the expression level of the protein GPX2 had also been reduced. As a member of the glutathione peroxidase system, GPX2 could reduce the content of hydroperoxide and play an important role in the body’s antioxidant defense (Gordon and Blobe, 2008). Therefore, the reduction of GPX2 after PM_2.5_ instillation might be related to oxidative stress caused by lung injury. In addition, LA was the precursor of AA, AA can be converted into PGE2 and leukotriene B4 (Čermák et al., 2016) to promote the development of inflammation. On the other hand, LA also had the effect of enhancing the immune function of the body. Metabolism of α-LA produced EPA, a congener of AA. EPA could compete with AA in the same enzyme system to produce PGE3 and inhibited the production of PGE2. Compared with PGE2, PGE3 did not promote the occurrence and development of inflammation in the inflammatory response, so α-LA played an anti-inflammatory effect in the body. The increase of α-LA and EPA after PM_2.5_ instillation might be a kind of compensatory self-protection machinery.

As a traditional Chinese prescription, YYQFD was often used to treat diphtheria, acute pharyngitis, and tonsillitis. As an exogenous pathogenic factor, PM_2.5_ instillation may lead to oxidative stress and inflammation in the lung. In this study, the lung injury mice model induced by PM_2.5_ was successfully established. Also, the effect and mechanism of YYQFD were evaluated and explored. We observed that the administration with YYQFD reduced the AA, 20-HETE, lecithin, PGE2, LA, α-LA, γ-LA and EPA levels in lung tissue. Meanwhile, PTGES2, CBR3 and GPX2 protein levels increased in lung tissue. Metabolomics and proteomics analysis revealed that YYQFD repaired lung injury caused by PM_2.5_ in mice by regulating arachidonic acid metabolism, linoleic acid metabolism, and biosynthesis of unsaturated fatty acids. These results suggested that AA, 20-HETE, lecithin, PGE2, LA, α-LA, γ-LA, EPA, PTGES2, CBR3, and GPX2 may be a putative drug target for improving PM_2.5_-induced pulmonary injury. Yet there are still some issues that should to be further investigated. In particular, the functional components of YYQFD are of warrant to be analyzed.

## Conclusion

Taken together, our results demonstrated that traditional Chinese prescription YYQFD significantly ameliorated PM_2.5_-induced lung damage by suppressing inflammatory factors and oxidative stress levels in the lung. The mechanism of YYQFD was mainly related to the reshaping of unsaturated fatty acid metabolism, characterized with the decreased levels of linoleic acid and its metabolite AA. The novel metabolic network map drawn in our study is favorable for a better understanding about the role of YYQFD on PM_2.5_-induced lung injury. Our study may provide a useful clue for the researchers targeting at the therapeutic role of YYQFD in lung diseases linked to air pollutants.

## Data Availability

The mass spectrometry proteomics data have been deposited to the ProteomeXchange Consortium (https://www.iprox.org/) *via* the PRIDE (Perez-Riverol et al., 2022) partner repository with the dataset identifier PXD037163.
