# Supplementary material for: Yangyinqingfei decoction attenuates PM2.5-induced lung injury by enhancing arachidonic acid metabolism
Source: Front Pharmacol. 2022 Nov 16;13:1056078. doi: 10.3389/fphar.2022.1056078 (PMC9708729; doi:10.3389/fphar.2022.1056078)
Supplement: Supplementary file 1 [file Table1.DOCX]

Table S1 Lung injury scoring system (Matute-Bello et al. 2011)

| Parameter | Score per field | | |
| --- | --- | --- | --- |
|  | 0 | 1 | 2 |
| 1. Neutrophils in the alveolar space | none | 1-5 | >5 |
| 1. Neutrophils in the interstitial space | none | 1-5 | >5 |
| 1. Hyaline membranes | none | 1 | >1 |
| 1. Proteinaceous debris filling the airspaces | none | 1 | >1 |
| 1. Alveolar septal thickening | <2**×** | 2**×**-4**×** | >4**×** |

Matute-Bello, G., Downey, G., Moore, B.B., Groshong, S.D., Matthay, M.A., Slutsky, A.S., et al. (2011). An official American Thoracic Society workshop report: features and measurements of experimental acute lung injury in animals. *Am. J. Respir. Cell Mol. Biol*. 44: 725-38. doi: 10.1165/rcmb.2009-0210ST.

Table S2 Metabolites with significant changes in mice exposed to PM_2.5_

| No. | m/z | *P*-value | VIP | Fold change | Name | KEGG.ID | Class | Ion mode |
| --- | --- | --- | --- | --- | --- | --- | --- | --- |
| 1 | 269.1596 | 0.000 | 1.133 | 8.13 | His-Ile |  |  | + |
| 2 | 184.0591 | 0.000 | 1.304 | 0.32 | 4-Pyridoxic acid | C00847 | Pyridinecarboxylic acids and derivatives | + |
| 3 | 568.3378 | 0.000 | 5.139 | 0.44 | 1-Stearoyl-sn-glycerol 3-phosphocholine |  |  | + |
| 4 | 186.1476 | 0.000 | 1.975 | 0.18 | Jasmine lactone |  |  | + |
| 5 | 171.0041 | 0.003 | 1.010 | 2.01 | Glyceraldehyde 3-phosphate | C00118 | Carbohydrates and carbohydrate conjugates | + |
| 6 | 298.2728 | 0.003 | 1.872 | 0.44 | Linoleic acid | C01595 | Lineolic acids and derivatives | + |
| 7 | 810.5970 | 0.007 | 10.991 | 0.36 | 1-Stearoyl-2-oleoyl-sn-glycerol 3-phosphocholine | C00157 | Glycerophosphocholines | + |
| 8 | 204.1223 | 0.008 | 3.203 | 0.60 | Acetylcarnitine | C02571 | Fatty acid esters | + |
| 9 | 522.3534 | 0.009 | 5.000 | 0.56 | 1-Oleoyl-sn-glycero-3-phosphocholine |  |  | + |
| 10 | 137.0704 | 0.017 | 1.604 | 0.66 | 1-Methylnicotinamide | C02918 | Pyridinecarboxylic acids and derivatives | + |
| 11 | 301.2150 | 0.030 | 1.903 | 1.89 | 4-Oxoretinol | C16683 | Retinoids | + |
| 12 | 627.5315 | 0.037 | 2.217 | 1.95 | 1-Stearoyl-2-arachidonoyl-sn-glycerol | C00165 |  | + |
| 13 | 400.3409 | 0.038 | 3.672 | 0.55 | L-Palmitoylcarnitine | C02990 | Fatty acid esters | + |
| 14 | 112.0499 | 0.039 | 2.499 | 1.93 | Cytosine | C00380 | Pyrimidines and pyrimidine derivatives | + |
| 15 | 76.0753 | 0.040 | 2.183 | 3.77 | Trimethylamine N-oxide | C01104 | Aminoxides | + |
| 16 | 468.3067 | 0.043 | 1.482 | 0.63 | 1-Myristoyl-sn-glycero-3-phosphocholine |  |  | + |
| 17 | 296.2576 | 0.049 | 4.719 | 0.36 | α-Linolenic acid | C06427 | Lineolic acids and derivatives | + |
| 18 | 215.1648 | 0.000 | 1.098 | 0.45 | 3-Hydroxydodecanoic acid |  | Medium-chain hydroxy acids and derivatives | - |
| 19 | 464.3111 | 0.002 | 2.289 | 70.00 | Glycocholic acid | C01921 | "Bile acids, alcohols and derivatives" | - |
| 20 | 303.2329 | 0.002 | 17.996 | 0.51 | Arachidonic Acid | C00219 | Fatty acids and conjugates | - |
| 21 | 338.9878 | 0.003 | 2.732 | 4.66 | D-Fructose 1,6-bisphosphate |  |  | - |
| 22 | 788.5424 | 0.003 | 2.341 | 2.10 | 2-Oleoyl-1-stearoyl-sn-glycero-3-phosphoserine |  |  | - |
| 23 | 639.4612 | 0.004 | 2.236 | 0.47 | 20-HETE |  |  | - |
| 24 | 105.0191 | 0.004 | 1.232 | 0.60 | Glyceric acid | C00258 | Carbohydrates and carbohydrate conjugates | - |
| 25 | 220.0822 | 0.005 | 1.331 | 0.44 | N-Acetylmannosamine | C00645 | Carbohydrates and carbohydrate conjugates | - |
| 26 | 273.0376 | 0.009 | 1.569 | 1.76 | 1-Deoxy-D-xylulose 5-phosphate | C11437 | Carbohydrates and carbohydrate conjugates | - |
| 27 | 322.0435 | 0.014 | 1.422 | 2.27 | 5'-CMP |  |  | - |
| 28 | 227.2018 | 0.022 | 2.842 | 0.80 | Myristic acid | C06424 | Fatty acids and conjugates | - |
| 29 | 465.3041 | 0.025 | 15.768 | 1.49 | Cholesterol 3-sulfate |  |  | - |
| 30 | 241.2167 | 0.029 | 1.118 | 0.80 | Pentadecanoic Acid | C16537 | Fatty acids and conjugates | - |
| 31 | 511.3490 | 0.031 | 2.891 | 1.36 | 3-Acetyl-11-keto-.beta.-boswellic acid |  |  | - |
| 32 | 313.2372 | 0.038 | 1.402 | 0.46 | 9,10-DiHOME |  |  | - |
| 33 | 180.0332 | 0.048 | 1.963 | 0.64 | Acamprosate |  | Organosulfonic acids and derivatives | - |

Note: control vs. model, Fold change>1, indicating that the metabolite was up-regulated in the model group; Fold change<1, indicating that the metabolite was down-regulated in the model group.

Table S3 Metabolites significantly changed by YYQFD intervention in PM_2.5_-induced lung injury mice

| No. | m/z | *P*-value | VIP | Fold change | Name | KEGG.ID | Class | Ion mode |
| --- | --- | --- | --- | --- | --- | --- | --- | --- |
| 1 | 100.1113 | 0.000 | 1.998 | 0.02 | Cyclohexylamine | C00571 | Cyclohexylamines | + |
| 2 | 113.0338 | 0.000 | 1.374 | 1.72 | Uracil | C00106 | Pyrimidines and pyrimidine derivatives | + |
| 3 | 175.1188 | 0.001 | 5.797 | 1.90 | L-Arginine | C00062 | "Amino acids, peptides, and analogues" | + |
| 4 | 303.2311 | 0.001 | 7.906 | 2.90 | Eicosapentaenoic acid | C06428 | Fatty acids and conjugates | + |
| 5 | 468.3067 | 0.001 | 1.805 | 1.63 | 1-Myristoyl-sn-glycero-3-phosphocholine |  |  | + |
| 6 | 522.3534 | 0.001 | 4.711 | 1.76 | 1-Oleoyl-sn-glycero-3-phosphocholine |  |  | + |
| 7 | 337.2365 | 0.002 | 1.260 | 2.92 | (2E,6E)-Farnesol |  |  | + |
| 8 | 120.0807 | 0.003 | 4.680 | 1.88 | Tyramine | C00483 | Phenethylamines | + |
| 9 | 123.0550 | 0.003 | 12.333 | 1.43 | Nicotinamide | C00153 | Pyridinecarboxylic acids and derivatives | + |
| 10 | 132.1013 | 0.003 | 1.994 | 1.87 | L-Isoleucine | C00407 | "Amino acids, peptides, and analogues" | + |
| 11 | 291.1292 | 0.005 | 1.331 | 2.14 | Argininosuccinic acid | C03406 | "Amino acids, peptides, and analogues" | + |
| 12 | 186.1476 | 0.005 | 1.083 | 2.19 | Jasmine lactone |  |  | + |
| 13 | 147.0759 | 0.005 | 2.348 | 1.34 | L-Pyroglutamic acid | C01879 | "Amino acids, peptides, and analogues" | + |
| 14 | 150.0575 | 0.005 | 1.719 | 2.05 | L-Methionine | C00073 | "Amino acids, peptides, and analogues" | + |
| 15 | 132.1012 | 0.006 | 2.593 | 1.74 | L-Leucine | C00123 | "Amino acids, peptides, and analogues" | + |
| 16 | 258.1099 | 0.007 | 7.875 | 1.94 | Glycerophosphocholine | C00670 | Glycerophosphocholines | + |
| 17 | 114.0904 | 0.010 | 1.221 | 1.48 | epsilon-Caprolactam |  |  | + |
| 18 | 298.2728 | 0.010 | 1.343 | 1.65 | Linoleic acid | C01595 | Lineolic acids and derivatives | + |
| 19 | 301.2150 | 0.011 | 1.829 | 2.40 | All-trans-retinoic acid (Vitamin A acid) | C00777 | Retinoids | + |
| 20 | 205.0957 | 0.011 | 1.307 | 2.08 | L-Tryptophan | C00078 | Indolyl carboxylic acids and derivatives | + |
| 21 | 370.2577 | 0.016 | 1.211 | 1.61 | Prostaglandin D2(PGD2) |  |  | + |
| 22 | 146.1174 | 0.017 | 2.738 | 1.35 | (3-Carboxypropyl)trimethylammonium cation |  |  | + |
| 23 | 204.1223 | 0.019 | 1.772 | 0.66 | Acetylcarnitine | C02571 | Fatty acid esters | + |
| 24 | 758.5667 | 0.022 | 5.574 | 1.77 | Thioetheramide-PC |  |  | + |
| 25 | 568.3378 | 0.023 | 3.224 | 1.64 | 1-Stearoyl-sn-glycerol 3-phosphocholine |  |  | + |
| 26 | 331.2620 | 0.028 | 1.938 | 5.83 | Eicosapentaenoic Acid ethyl ester |  |  | + |
| 27 | 130.0856 | 0.028 | 1.704 | 1.66 | D-Pipecolinic acid |  |  | + |
| 28 | 319.2257 | 0.037 | 1.547 | 2.01 | 12-oxo-ETE |  |  | + |
| 29 | 296.2576 | 0.040 | 3.440 | 2.37 | α-Linolenic acid | C06427 | Lineolic acids and derivatives | + |
| 30 | 469.3375 | 0.000 | 1.362 | 0.02 | 11-Keto-.beta.-boswellic acid |  |  | - |
| 31 | 233.1542 | 0.000 | 3.521 | 1.72 | Confertifoline |  |  | - |
| 32 | 511.3490 | 0.000 | 30.744 | 1.90 | 3-Acetyl-11-keto-.beta.-boswellic acid |  |  | - |
| 33 | 227.2018 | 0.000 | 21.012 | 2.90 | Myristic acid | C06424 | Fatty acids and conjugates | - |
| 34 | 277.2164 | 0.000 | 4.211 | 1.63 | all cis-(6,9,12)-Linolenic acid |  |  | - |
| 35 | 369.2276 | 0.002 | 2.037 | 1.76 | 6-Keto-PGF1a |  |  | - |
| 36 | 111.0204 | 0.002 | 3.940 | 2.92 | Uracil | C00106 | Pyrimidines and pyrimidine derivatives | - |
| 37 | 241.2167 | 0.002 | 1.628 | 1.88 | Pentadecanoic Acid | C16537 | Fatty acids and conjugates | - |
| 38 | 116.0716 | 0.004 | 2.197 | 1.43 | L-Valine | C00183 | "Amino acids, peptides, and analogues" | - |
| 39 | 114.0560 | 0.004 | 1.597 | 1.87 | D-Proline | C00763 | "Amino acids, peptides, and analogues" | - |
| 40 | 145.0979 | 0.006 | 1.267 | 2.14 | L-Lysine | C00047 | "Amino acids, peptides, and analogues" | - |
| 41 | 313.2372 | 0.012 | 1.115 | 2.19 | 9,10-DiHOME |  |  | - |
| 42 | 164.0718 | 0.012 | 3.181 | 1.34 | L-Phenylalanine | C00079 | "Amino acids, peptides, and analogues" | - |
| 43 | 130.0874 | 0.017 | 2.318 | 2.05 | L-Isoleucine | C00407 | "Amino acids, peptides, and analogues" | - |
| 44 | 130.0877 | 0.018 | 4.014 | 1.74 | L-Leucine | C00123 | "Amino acids, peptides, and analogues" | - |
| 45 | 339.1998 | 0.019 | 3.803 | 1.94 | Norethindrone Acetate |  |  | - |
| 46 | 303.2329 | 0.023 | 9.647 | 1.48 | Arachidonic Acid | C00219 | Fatty acids and conjugates | - |
| 47 | 242.0796 | 0.023 | 1.495 | 1.65 | Phosphorylcholine | C00588 | Quaternary ammonium salts | - |
| 48 | 293.2111 | 0.025 | 1.868 | 2.40 | 13-OxoODE |  |  | - |
| 49 | 179.0560 | 0.025 | 3.203 | 2.08 | Alpha-D-Glucose | C00267 | Carbohydrates and carbohydrate conjugates | - |
| 50 | 180.0664 | 0.034 | 1.183 | 1.61 | L-Tyrosine | C00082 |  | - |
| 51 | 351.2168 | 0.044 | 3.366 | 1.35 | Prostaglandin E2 | C00584 | Eicosanoids | - |

Note: control vs. model, Fold change>1, indicating that the metabolite was up-regulated in the model group; Fold change<1, indicating that the metabolite was down-regulated in the model group.

Table S4 Proteins with significant changes in mice exposed to PM_2.5_

| No. | Protein name | Gene name | *P*-value | Fold change |
| --- | --- | --- | --- | --- |
| 1 | Uncharacterized protein | Bcl11b | 0.003 | 2.06 |
| 2 | Single-pass membrane and coiled-coil domain-containing protein 4 | Smco4 | 0.014 | 2.03 |
| 3 | Protein S100-A11 | S100a11 | 0.010 | 1.86 |
| 4 | Podoplanin | Pdpn | 0.002 | 1.82 |
| 5 | Differentially expressed in FDCP 6 | Def6 | 0.015 | 1.77 |
| 6 | Histone H3.3 | H3-3a | 0.015 | 1.64 |
| 7 | Tropomyosin alpha-1 chain | Tpm1 | 0.005 | 1.64 |
| 8 | Tpm3 protein | Tpm3 | 0.000 | 1.63 |
| 9 | Tropomyosin alpha-4 chain | Tpm4 | 0.019 | 1.61 |
| 10 | H15 domain-containing protein | H1f3 | 0.039 | 1.60 |
| 11 | Beta-globin | Hbbt1 | 0.019 | 1.58 |
| 12 | Hematopoietic progenitor cell antigen CD34 | Cd34 | 0.010 | 1.58 |
| 13 | Apolipoprotein C-I | Apoc1 | 0.010 | 1.57 |
| 14 | Acyl-coenzyme A synthetase ACSM2, mitochondrial | Acsm2 | 0.038 | 1.54 |
| 15 | Dynein heavy chain 5, axonemal | Dnah5 | 0.001 | 1.54 |
| 16 | Protein eva-1 homolog B | Eva1b | 0.007 | 1.53 |
| 17 | Serine/threonine-protein kinase PLK | Plk1 | 0.001 | 1.53 |
| 18 | Histone H1.2 | H1-2 | 0.021 | 1.53 |
| 19 | Histone H2A (Fragment) | H2A | 0.008 | 1.52 |
| 20 | Vesicle-associated membrane protein 8 | Vamp8 | 0.025 | 1.51 |
| 21 | Transmembrane protein 109 | Tmem109 | 0.001 | 1.51 |
| 22 | Histone H2B type 3-A | Hist3h2ba | 0.034 | 1.51 |
| 23 | Zinc finger protein 511 (Fragment) | Zfp511 | 0.019 | 0.66 |
| 24 | Serpin B5 | Serpinb5 | 0.048 | 0.66 |
| 25 | Probable lysosomal cobalamin transporter | Lmbrd1 | 0.003 | 0.66 |
| 26 | Uncharacterized protein | 5330429B09Rik | 0.016 | 0.65 |
| 27 | Rho-GAP domain-containing protein | Arhgap6 | 0.002 | 0.64 |
| 28 | Alpha-ketoglutarate-dependent dioxygenase FTO | Fto | 0.005 | 0.63 |
| 29 | Protease, serine 1 (trypsin 1) | Prss1 | 0.000 | 0.62 |
| 30 | Dehydrogenase/reductase SDR family member on chromosome X homolog | Dhrsx | 0.043 | 0.62 |
| 31 | Protein SET (Fragment) | Set | 0.040 | 0.61 |
| 32 | Zinc finger protein 513 (Fragment) | Zfp513 | 0.020 | 0.61 |
| 33 | LAM_G_DOMAIN domain-containing protein | Col9a1 | 0.026 | 0.59 |
| 34 | Potassium voltage-gated channel subfamily KQT member 5 | Kcnq5 | 0.006 | 0.59 |
| 35 | Proteasome adapter and scaffold protein ECM29 | Ecpas | 0.003 | 0.58 |
| 36 | Serine/arginine repetitive matrix protein 1 (Fragment) | Srrm1 | 0.002 | 0.57 |
| 37 | RIKEN cDNA 1700074P13 gene (Fragment) | 1700074P13Rik | 0.007 | 0.57 |
| 38 | Mediator of RNA polymerase II transcription subunit 1 | Med1 | 0.013 | 0.57 |
| 39 | Try10-like trypsinogen | Gm5409 | 0.000 | 0.57 |
| 40 | Peptidase S1 domain-containing protein | Gzma | 0.034 | 0.56 |
| 41 | Bms1-type G domain-containing protein (Fragment) | Bms1 | 0.007 | 0.56 |
| 42 | Peptidase S1 domain-containing protein | Try5 | 0.003 | 0.55 |
| 43 | ATP synthase protein 8 | ATP8 | 0.024 | 0.54 |
| 44 | Probable 28S rRNA (cytosine-C(5))-methyltransferase | Nop2 | 0.043 | 0.52 |
| 45 | Gamma-parvin | Parvg | 0.000 | 0.52 |
| 46 | Transcription initiation factor TFIID subunit 5 | Taf5 | 0.004 | 0.49 |
| 47 | Meiosis regulator and mRNA stability factor 1 | Marf1 | 0.036 | 0.25 |
| 48 | Ryanodine receptor 1 | Ryr1 | 0.000 | 0.04 |

Note: control vs. model, Fold change>1, indicating that the protein was up-regulated in the model group; Fold change<1, indicating that the protein was down-regulated in the model group.

Table S5 Proteins significantly changed by YYQFD intervention in PM_2.5_-induced lung injury mice

| No. | Protein name | Gene name | *P*-value | Fold change |
| --- | --- | --- | --- | --- |
| 1 | Vps16_C domain-containing protein | Vipas39 | 0.009 | 1.86 |
| 2 | E3 ubiquitin-protein ligase Jade-2 | Jade2 | 0.002 | 1.70 |
| 3 | ANK_REP_REGION domain-containing protein | Gabpb1 | 0.015 | 1.65 |
| 4 | Ribosome-binding protein 1 | Rrbp1 | 0.014 | 1.59 |
| 5 | Proteasomal ubiquitin receptor ADRM1 | Adrm1 | 0.005 | 1.58 |
| 6 | Uncharacterized protein (Fragment) | Gabpb2 | 0.012 | 1.58 |
| 7 | Hmgn2 protein | Hmgn2 | 0.013 | 1.58 |
| 8 | Rho GTPase-activating protein 39 | Arhgap39 | 0.033 | 1.54 |
| 9 | Myozenin-2 | Myoz2 | 0.045 | 0.66 |
| 10 | Myosin light chain 3 | Myl3 | 0.004 | 0.65 |
| 11 | Calsequestrin-1 | Casq1 | 0.048 | 0.63 |
| 12 | Cysteine and glycine-rich protein 3 | Csrp3 | 0.001 | 0.62 |
| 13 | Sarcalumenin | Srl | 0.033 | 0.62 |
| 14 | Cytochrome c oxidase subunit 7A1, mitochondrial | Cox7a1 | 0.033 | 0.62 |
| 15 | Creatine kinase M-type | Ckm | 0.028 | 0.59 |
| 16 | MICOS complex subunit MIC60 | Immt | 0.034 | 0.59 |
| 17 | Inactive serine/threonine-protein kinase VRK3 (Fragment) | Vrk3 | 0.039 | 0.53 |
| 18 | Caveolin (Fragment) | Cav1 | 0.005 | 0.51 |
| 19 | PCI domain-containing protein | Cops7b | 0.006 | 0.50 |
| 20 | Tropomyosin alpha-1 chain | Vipas39 | 0.004 | 0.40 |

Note: control vs. model, Fold change>1, indicating that the protein was up-regulated in the model group; Fold change<1, indicating that the protein was down-regulated in the model group.
